# Novel Insights into Obesity in Preschool Children with Autism Spectrum Disorder

**DOI:** 10.1007/s10578-024-01679-1

**Published:** 2024-02-01

**Authors:** Anna van der Lubbe, Hanna Swaab, Robert Vermeiren, Erica van den Akker, Wietske Ester

**Affiliations:** 1 Sarr Autism Rotterdam, Youz Child- and Adolescent Psychiatry, Parnassia Group, Dynamostraat 18, Rotterdam, 3083 AK The Netherlands; 2 Clinical Neurodevelopmental Sciences, Leiden University, Wassenaarseweg 52, Leiden, The Netherlands; 3 Parnassia Group, Parnassia Academy, Den Haag, The Netherlands; 4 Child- and Adolescent Psychiatry, LUMC-Curium, Endegeesterstraatweg 27, Oegstgeest, The Netherlands; 5 Leiden Institute for Brain and Cognition, Leiden University, Leiden, The Netherlands; 6 Department of Pediatrics, Division Pediatric Endocrinology and Obesity Center CGG NL, Erasmus University Medical Centre-Sophia Children’s Hospital, Dr. Molewaterplein 40, Rotterdam, The Netherlands

**Keywords:** Obesity, Autism, Preschoolers, Eating behavior

## Abstract

**Abstract:**

Obesity is present in 8–32% of the children with Autism Spectrum Disorder (ASD). However, most studies are performed in school-aged children from the USA. The current study compares obesity rates of Dutch preschoolers with ASD with children from the Dutch general population and explores which child- and parental factors are related to obesity in children with ASD. This cross-sectional study is part of the ongoing Tandem Study (Dutch Trial register: NL7534). Seventy-eight children with ASD aged 3–7 years and their parents (77 mothers, 67 fathers) participated. Child factors are: Body Mass Index (by physical measurement), child eating behavior (Child Eating Behavior Questionnaire), child problem behavior (Child Behavior Checklist), and ASD severity (Autism Diagnostic Observation Scale 2). Parental factors are: BMI (by physical measurement), parental eating behavior (Dutch Eating Behavior Inventory), parenting stress (The Parenting Stress Questionnaire) and highest completed educational level (SES). Children with ASD were 8 times more often obese (16.8%) than children from the general population (2.0%). Child BMI correlated positively with child food approach behavior and maternal BMI, and correlated negatively with child ‘Slowness in eating’. There was no correlation between child BMI and ASD severity, problem behavior, parental eating behavior, parental stress and SES. Thus, Dutch, preschool children with ASD have 8 times higher obesity rates than children from the general population. More attention to obesity risk in research and clinical care could contribute to the quality of life of individuals with ASD and their families.

**Clinical Trial Registration:**

Dutch Trial register, NL7534, https://trialsearch.who.int/Trial2.aspx?TrialID=NL7534.

**Supplementary Information:**

The online version contains supplementary material available at 10.1007/s10578-024-01679-1.

Previous studies show that individuals with Autism Spectrum Disorder (ASD) may have a two to three times higher risk for morbidity and early mortality than individuals from the general population [1, 2]. One condition that has frequently been associated with morbidity and mortality is obesity. A Swedish population study (n = 41,359) demonstrated that individuals who were obese during childhood, had a three times higher risk for mortality during early adulthood compared to same-aged individuals from the general population (0.55% vs. 0.19%) [3]. Moreover, other studies in non-ASD populations have associated childhood obesity with high life-time risk for various chronic conditions, including diabetes, multiple types of cancer, cardiovascular disease and adult obesity [2, 4–6]. Research suggests that childhood obesity is preventable and treatable [7]. Therefore, it may be particularly important to focus on childhood obesity to understand and possibly prevent health problems in individuals with ASD.

Recent meta-analyses indicate that children and adolescents with ASD have higher prevalence rates of obesity, ranging from 7.9 to 31.8% compared to 1.4–23.6% among individuals without ASD [8, 9]. Learning more about obesity in individuals with ASD is relevant from several perspectives. First, high obesity rates can be considered as a risk factor for future health problems. From this perspective, research focusing on obesity may support the development of future prevention strategies of health problems in children with ASD. Second, it could be theorized that a high risk for obesity reflects a genetic disposition that may put individuals with ASD at higher risk for obesity and other health problems as well. For example, some genomic imbalances that have been associated with ASD have also been associated with childhood obesity [10]. From this point of view, research focusing on obesity in early childhood may increase the understanding of pathways to physical health problems in general in individuals with ASD by identifying possible vulnerability already at an early age.

While overweight and obesity in children with ASD have been investigated before, most studies so far have focused on obesity in children of a broad age-range, mostly school- age. Early childhood may be a particularly relevant period to study obesity, since early development may impact the risk for obesity and other health problems in later childhood, adolescence and adulthood [11]. In addition, most studies have been performed in the United States. Since childhood obesity is more prevalent in the United States than in other Western countries, it could be argued that the prevalence rates of obesity in children with ASD might be different in other countries [12].

Previous studies have demonstrated several factors associated to overweight or obesity in children with ASD, such as lower parental education levels and sleep problems [13]. However, again, most studies focused on older children with ASD or children in a broad age-range. Furthermore, less attention has been directed at parental health factors that may be related to their child’s health. This may be particularly important for children with ASD, as a recent study demonstrated higher rates of obesity in mothers of children with ASD and higher rates of clinical parenting stress in both mothers and fathers of a child with ASD compared to the general population [14]. This may be relevant for the health of their children, as previous studies have associated parenting stress and parental obesity with the risk for childhood obesity [15, 16].

The first goal of the current study is to investigate whether Dutch, preschool children with ASD show higher obesity rates compared to children from the general Dutch population. The second goal of the study is to explore which child factors (ASD severity, eating behavior and problem behavior) and parental factors (eating behavior, BMI, parenting stress, demographics) are associated with obesity in Dutch, preschool children with ASD.

## Method

### Procedure

The current study is a cross-sectional study investigating obesity in preschool children with ASD. This study is part of the ongoing Tandem Study (Dutch Trial register: NL7534), approved by the Institutional Review Board of the Leiden University Medical Center, The Netherlands. Data were collected between 2018 and 2022.

### Participants

Families were recruited from Youz Child and Adolescent Psychiatry (Parnassia Group), GGZ Delfland and Jonx, which are large mental health care providers with multiple locations throughout the west, middle or north of the Netherlands. Families were eligible for inclusion if: (1) the child was diagnosed with ASD, (2) the child was aged between 3 and 7 years and (3) parents could understand Dutch or English without the help of a translator. Children who started new psychotropic medication three months prior to participating in the study were excluded. If parents were eligible for inclusion and agreed to be contacted by the research team, parents received an oral and written description of the study. If parents decided to participate in the study, they met with a researcher to complete the informed consent process.

### Measures

#### Obesity

Body height was measured using a stadiometer (Seca 213), and body weight by a digital scale (Seca Clara 803) in all participants. Body Mass Index (BMI) was calculated by dividing weight in kilograms by the square of height in meters. Child BMI was standardized to BMIz, using Growth Analyser Software Research Calculation Tools version 4.1.5 with the Fifth National Dutch Growth Study as a reference group. Based on international cut-off points by Cole and colleagues, children were classified into three BMI classes: healthy weight, overweight and obese [17]. The percentage of participants in each category was compared to Dutch children aged 2–21 years (n = 20.867) from the Fifth Dutch Growth Study, the actual standard of comparison in Dutch pediatric health care [18]. In addition to physical measurements, parents also reported their own height and weight as part of the Dutch Eating Behavior Questionnaire.

#### ASD Severity

ASD severity was measured using the Autism Diagnostic Observation Scale (ADOS-2) [19]. The ADOS-2 is a standardized, semi-structured observational measure of ASD symptoms. For this study, we used the standardized ADOS severity score, ranging from 0 (minimal) to 10 (high), representing the severity of autism symptoms.

#### Children’s Eating Behavior

Child eating behavior was measured using the Child Eating Behavior Questionnaire (CEBQ). The CEBQ is a 35-item questionnaire consisting of 8 subscales measuring food approach behaviors (subscales: Food Responsiveness, Enjoyment of Food, Emotional Overeating and Desire to Drink) and food avoidant behaviors (subscales: Satiety Responsiveness, Slowness in Eating, Emotional Under-Eating and Food Fussiness). Mothers rated items on a 5-point Likert scale, with higher scores indicating a higher level of the specific behavior. The CEBQ has good psychometric properties in terms of factor structure, internal reliability and correlations between subscales [20]. Cronbach’s alpha values for the subscales range from 0.75 to 0.91 [21].

#### Behavior Problems

Behavior problems were measured using the Child Behavior Checklist (CBCL) [22, 23] The CBCL is a caregiver report form targeting problem behavior in children, using two versions: the preschool version (CBCL/1½-5), containing 100 problem behavior questions and the school-age version (CBCL/6–18), containing 118 problem behavior questions. Mothers rated their child’s problem behavior on a 3-point scale, with higher scores reflecting a higher level of the corresponding behavior. For the current study, the total raw score and the raw scores on the subscales Internalizing and Externalizing problems were used. As both versions have a different number of items, the total raw score on each subscale was divided by the number of items for comparability between the two versions.

#### Parental Eating Behavior

Parental eating behavior was measured using the Dutch Eating Behavior Questionnaire (DEBQ) [24]. The DEBQ is a 33-item self-report measure of eating behavior consisting of 3 subscales: Emotional eating, External eating and Restrained eating. Items are scored on 5-point Likert scale. Higher subscale scores indicate a higher level of the corresponding specific eating behavior. Cronbach’s alpha value is 0.96 for Emotional eating, > 0.78 for External eating and > 0.90 for Restrained eating.

#### Parenting Stress

Parenting stress was measured using the Parenting Stress Questionnaire (OBVL). The OBVL is a 34-item self-report measure of parenting stress [25]. Items are answered on a 4-point Likert scale. For this study, the total score on the OBVL was used (*α* **=** 0.91), in which a high score reflects a high level of parenting stress.

#### Demographic and Descriptive Variables

Parents indicated their highest completed education and their birth country. The highest completed education of mother was used as a measure of Social Economic Status (SES). To be consistent with our comparison group, the participants of the Fifth Dutch National Growth Study, an indication of ethnic background of the child was derived based on the birth country of parents. Children were categorized into one of the two categories: (1) Non migration background and (2) Migration background (if one- or both parents was born outside the Netherlands). Parents also reported their child’s medication use and whether their children had any other physical or psychiatric health problems. Parents filled in their marital status (married/cohabiting versus single parent), the primary caregiver and whether they had paid employment.

Adaptive functioning was measured using the Vineland Screener 0–6 years (Vineland-S). The Vineland-S is a 72-item questionnaire reflecting the level of adaptive functioning of children aged 0 to 6 years in four subscales, including communication, social abilities, daily living skills and motor skills [26]. The Vineland-S has been developed and validated in the Netherlands for children aged 0 to 6 years and for older children with a developmental age corresponding to the calendar age range of the Vineland Screener. For our study, we used the total score to express the level of adaptive functioning.

### Statistical Analyses

We used a chi-square test of independence to determine whether the proportion of children scoring above the previously mentioned weight cut-offs was different from the reference population. As the reference population consisted only of Dutch children without a migration background, we performed an additional analysis excluding the children in our sample with a migration background to test whether this would affect our results. Moreover, we performed an additional analysis excluding the children who used appetite-inducing medication.

To explore which factors are associated with child BMIz in children with ASD, we performed a Pearson’s or Spearman’s correlation analysis, depending on the (normal) distribution of the variables. In addition to zero-order correlations, partial correlation coefficients were also calculated controlling for SES and ethnic background.

If BMI values were missing in children, BMI values were collected during the next visit 6 months later. If physical height or weight data was missing in fathers and mothers, parental self-reported height and weight measures were used. If total scores or subscale scores were missing, pairwise deletion in analyses was used. All analyses were performed in SPSS Statistics 25.

## Results

### Descriptives

Seventy-eight children with ASD aged 3 to 7 years (Median = 5.2; Interquartile Range [IQR] = 2.2) and their parents (77 mothers, 67 fathers) participated. The demographic characteristics of the children are displayed in Table 1. Autism severity (ADOS) scores ranged from 1 to 10 (Median = 7, IQR = 3). Regarding CBCL scores, 22.2% of the children scored within normal range, 11.1% scored within the subclinical range and 66.7% scored within the clinical range of behavioral problems. Adaptive functioning was low (below 25th percentile on the Vineland screener) in 83.3% of the children, average (between 25th and 75th percentile) in 13.9% of the children and high (above 75th percentile) in 2.8% of the children. Medication used is listed in Table S1. There was one child in our sample with a sex chromosome abnormality, the rest of our sample did not have any (known) genetic disorder. To our knowledge, one mother was pregnant during the study. Excluding her from analysis did not affect our results. Data was collected between November 2018 and March 2023.

**Table 1:** Demographic and descriptive characteristics of children with ASD aged 3–7 years (n = 78)

		n	%
Child gender			
	Boy	64	82.1
	Girl	14	17.9
Child use of medication			
	Yes, appetite inducing medication	7	12.5
	Yes, appetite reducing medication	1	1.8
	Yes, but without effect on appetite	3	5.4
	No	55	77.5
Highest completed educational level of mother			
	Primary school	1	1.4
	Lower vocational secondary education	5	7.2
	Lower secondary education	1	1.4
	Intermediate vocational education	29	42.0
	Intermediate secondary education	2	2.9
	Higher secondary education	1	1.4
	Higher vocational education	22	31.9
	University	8	11.6
Ethnic background of the child^a^			
	Dutch	47	64.4
	Migration background	26	35.6
Autism Severity scores (ADOS-2)			
	Low to minimum	16	22.9
	Moderate	30	42.9
	High	24	34.3
Externalizing behavior problems (CBCL)			
	Normal range	25	34.7
	Subclinical range	5	6.9
	Clinical range	42	58.3
Internalizing behavior problems (CBCL)			
	Normal range	18	25.0
	Subclinical range	5	6.9
	Clinical range	49	68.1
Total behavior problems (CBCL)			
	Normal range	16	22.2
	Subclinical range	8	11.1
	Clinical range	48	66.7
Adaptive functioning (Vineland screener 0–6)			
	Low (< 25th percentile)	60	83.3
	Average (between 25th and 75th percentile)	12	13.9
	High (above 75th percentile)	2	2.8

^a^Children were classified with a migration background if one or more parents were born in a different country than the Netherlands

As three children refused physical measurements, the missing BMI values were replaced by child BMI values that were measured during the next visit 6 months later (*r* = .89, *p* = < 0.001). These children did not use medication. Parental height was missing in 3 parents and parental weight was missing in 1 parent and were replaced by self-reported height (*r* = .97, *p* < .001) and weight (*r* = .99, *p* < .001).

Parents were married or co-habiting in 57 families (73.1%), the parent was a single parent in 16 of the families (20.5%) and marital status was missing in 6 families (6.4%). In total, 78.3% of the mothers and 95.7% of the fathers had paid employment.

### Obesity Rates

As shown in Table 2, almost 17% of the children with ASD were obese, which is more than 8 times higher than the rates of obesity (2%) of Dutch children from the Fifth National Growth Study (*χ*^*2*^*(3)* = *81.5*, *p* < .001). This difference remained significant after excluding children using medication or children from a migration background (see Table S2). Furthermore, 9% of the children with ASD were overweight, while the national prevalence rate is about 12%.

**Table 2:** Overweight and obesity in children with ASD (3–7 years) compared to Dutch children aged 2–21 years from the Fifth National Growth Study (Schonbeck & van Buuren, 2010)

	Children with ASD (n = 78)		Reference group (n = 12,151)		
	N	%	%	Chi-square	*p*
Not overweight	58	74.4	85.9	81.5	< 0.001
Overweight	7	9.0	12.1		
Obesity	13	16.7	2		

### Correlations between Body Mass Index, Child- and Parental Factors

#### Child Factors

As displayed in Table 3, a higher BMIz was related to more food responsiveness (*r =* .43, *p* < .001), emotional overeating (*r =* .30, *p* = .011), enjoyment of food (*r =* .36, *p* = .002) and desire to drink (*r =* .28, *p* = .019) in children with ASD. In addition, child BMIz correlated negatively with slowness in eating (*r = −* .32, *p* = .006). There was no significant correlation between child BMIz and the other food avoidance scales of the CEBQ. Child BMIz did not correlate significantly with autism severity and behavior problems. All correlations remained significant after controlling for SES and ethnic background (see Table S3).

**Table 3:** Correlations between BMIz of children with ASD and autism severity, eating behavior and behavior problems

	BMIz Child 3–7 years of age
*Autism severity (ADOS-2)*	
Autism Severity^a^	− 0.08
*Child eating behavior (CEBQ)*	
Food responsiveness^a^	0.43***
Emotional overeating^a^	0.30*
Enjoyment of food	0.36**
Desire to drink	0.30*
Satiety responsiveness	− 0.23
Slowness in eating	− 0.32**
Emotional undereating^a^	− 0.10
Food fussiness^a^	− 0.04
*Child problem behavior (CBCL)*	
Externalizing behavior problems	0.10
Internalizing behavior problems	0.09
Total behavior problems	0.22

*Abbreviations* BMIz = Standardized Body Mass Index; ASD = Autism Spectrum Disorder; ADOS = Autism Diagnostic Observation Scale; CEBQ = Child Eating Behavior Questionnaire; CBCL = Child Behavior Checklist. ^a^Variable was non-normally distributed, Spearman’s correlation coefficients are displayed. *p < .05, **p < .01, ***p < .001

#### Parental Factors

As presented in Table 4, there was a significant correlation between child BMIz and mother’s BMI (*r =* .29, *p* = .011), which remained significant after controlling for SES and ethnic background (see Table S4). Child BMIz did not correlate significantly with father’s BMI, parental eating behavior, parenting stress and parental educational level.

**Table 4:** Correlations between BMI of children with ASD and parental BMI, eating behavior and SES

	BMI Child
*Mothers*	
BMI^a^	0.29*
Emotional eating (DEBQ)^a^	0.05
External eating (DEBQ)	0.05
Restraint eating (DEBQ)^a^	0.19
Parenting stress (OBVL)	0.04
Highest completed education^a^	− 0.17
*Fathers*	
BMI^a^	0.20
Emotional eating (DEBQ)^a^	0.04
External eating (DEBQ)	− 0.21
Restraint eating (DEBQ)^a^	− 0.20
Parenting stress (OBVL)	− 0.01
Highest completed education^a^	− 0.15

*Abbreviations* ASD = Autism Spectrum Disorder; BMI = Body Mass Index; SES = Social Economic Status; DEBQ=Dutch Eating Behavior Questionnaire; OBVL = Parenting Stress Questionnaire; SES = Social Economic Status. ^a^Variable was non-normally distributed, Spearman’s correlation coefficients are displayed. *p < .05, **p < .01, ***p < .001

## Discussion

The current study investigated rates of obesity in preschool children with ASD living in the Netherlands and explored several possible contributing child and parental factors. Almost 17% of the children with ASD was obese, which is more than 8 times higher than the national prevalence rates of childhood obesity (2%). Children with a higher BMI showed more food approach behavior and less slowness in eating. Child BMI correlated positively with maternal BMI. We did not find an association between child BMI, child problem behavior, autism severity, parental disinhibited eating behavior, BMI of fathers and SES.

The higher rates of obesity in children with ASD compared to the general population is in line with previous studies from the USA [9]. On the contrary, none of the European studies that were included in the meta-analysis of Sammels and colleagues found a significant difference between the obesity rates of children with ASD and those of non-ASD individuals [22, 27–29]. They propose several explanations for this, such as the use of a psychiatric control group and inadequate power. However, obesity rates in our study are in line with age-specific obesity rates as reported by the meta-analysis of Li and colleagues [30], reporting obesity in 16.7% of the children with ASD between 2 and 5 of age.

We found an association between BMI and food approach behavior in children with ASD, which is in line with earlier studies in neurotypical children [20]. This association may be particularly important for children with ASD, as previous studies suggest that compared to their neurotypical peers, children with ASD are more likely to engage in food approach behavior, including emotional overeating [31, 32]. We did not find an association between BMI and autism severity or child problem behavior. Previous studies that have investigated the association between BMI and autism severity displayed contrasting results. While some studies associated autism severity with higher odds of being overweight in children and adolescents with ASD, some studies found an inverse relationship between autism severity and BMI in girls with ASD, or no association [13, 28, 33–35]. Therefore, we encourage future studies to further investigate this association by including possible moderating factors, such as gender or age-group.

Children with a higher BMI had mothers with a higher BMI, also after controlling for SES and ethnicity. This is in line with an earlier American study, indicating parental obesity as a strong predictor for obesity in children with ASD [36]. Possible explanations for this are genetic susceptibility, shared environment, or a combination of both. Child BMI was not associated with the educational level of their parents, which contrasts with international studies that observe a negative relationship between SES and the prevalence of childhood BMI in high-income countries [37]. Therefore, the high levels of obesity in our sample may reflect a health-risk that is specific for (families of) children with ASD.

The current study had some limitations. We used a national reference group to compare obesity rates. One might consider this a limitation, as the Fifth National Growth study did not include children with a migration background and in our study population 36% of the ASD children had a migration background. However, we showed that obesity rates in our population remained significantly higher than the comparison group after excluding children with ASD with a migration background, indicating the robustness of the finding. In addition, there was a difference between comparison samples in age-range, as our comparison group was aged between 2 and 21 years, while our sample was aged between 3 and 7 years. However, Schönbeck and colleagues [18] reported age-specific obesity rates of our reference group that range from 0.8 to 3.4% in children aged 3–6 years, which is 5–21 times lower than the obesity rates we found in children with ASD. Therefore, we think it is likely that observed differences cannot be attributed to age differences. We consider it a strength of the study that we used an integrated approach, in which concurrently mental- and physical measures were examined of both parents and children. Furthermore, all children that participated in this study were between 3 and 7 years old and recently received an ASD diagnosis, which allowed us to investigate obesity during an early developmental stage.

## Summary

The current study evaluated obesity rates in preschool children with ASD and explored possible factors associated with obesity. Almost 17% of the children in our study was obese, which is more than 8 times higher than in the Dutch general population. Moreover, almost 9% of the children with ASD were at risk for obesity and classified as overweight. Children with a higher BMI showed more food approach behavior and less slow eating behavior. In addition, children with a higher BMI had mothers with a higher BMI. We did not find an significant association between child BMI and ASD severity, problem behavior, parental eating behavior, parental stress and SES.

To better understand underlying mechanisms, more research is needed. As childhood obesity can profoundly affect children’s physical- and psychological well-being, it is important to target obesity and obesity related behavioral factors like food approach behavior in the treatment of ASD. We encourage professionals to screen for (risk for) obesity during standard clinical care of individuals with ASD. Some studies have demonstrated weight loss after intervention in children with ASD, which suggests that improvement is possible [29]. However, it would be even better to prevent obesity by educating parents of young children with ASD about the risk for obesity and the associated health risks and guiding them to a healthy lifestyle, as breaking habits is more difficult than learning healthy patterns right away.

## Electronic Supplementary Material

Below is the link to the electronic supplementary material.

Supplementary Material 1

Supplementary Material 2

Supplementary Material 3

Supplementary Material 4

## Data Availability

Not applicable.
