# Supplementary material for: Novel Insights into Obesity in Preschool Children with Autism Spectrum Disorder
Source: Child Psychiatry Hum Dev. 2024 Feb 1;57(1):2–9. doi: 10.1007/s10578-024-01679-1 (PMC12971737; doi:10.1007/s10578-024-01679-1)
Supplement: Supplementary file 1 — Supplementary Material 1 [file 10578_2024_1679_MOESM1_ESM.docx]

| Table S1.  Medications taken by children with ASD aged 3 – 7 years (n = 16). | | |
| --- | --- | --- |
| Medication name | | N |
| *Appetite inducing medication* | |  |
| Anti-epileptics | |  |
|  | Valproic acid | 2 |
| Antihistamines | |  |
|  | Desloratadine | 2 |
|  | Yes, name of medicine not specified | 2 |
| Antipsychotics | |  |
|  | Aripiprazole | 3 |
| Corticosteroids | |  |
|  | Dexamethasone | 1 |
|  | Triamcinolone Acetonide Cream | 1 |
|  | Hydrocortisone cream | 1 |
| *Appetite reducing medication* | |  |
| Amphetamines | |  |
|  | Methylphenidate | 1 |
|  | Dexamphetamine | 1 |
| *Medication without effect on appetite* | |  |
| Acetanilide Derivative | |  |
|  | Paracetamol | 1 |
| Benzodiazepine | |  |
|  | Midazolam | 1 |
|  | Clobazam | 1 |
| Beta-2 adrenergic receptor agonist | |  |
|  | Ventolin | 3 |
| Leukotriene receptor antagonists | |  |
|  | Montelukast | 1 |
| Metatonin receptor agonists | |  |
|  | Melatonin | 1 |
| Other | |  |
|  | Vaseline cetomacrogol cream | 1 |
