# Supplementary material for: Novel Insights into Obesity in Preschool Children with Autism Spectrum Disorder
Source: Child Psychiatry Hum Dev. 2024 Feb 1;57(1):2–9. doi: 10.1007/s10578-024-01679-1 (PMC12971737; doi:10.1007/s10578-024-01679-1)
Supplement: Supplementary file 2 — Supplementary Material 2 [file 10578_2024_1679_MOESM2_ESM.docx]

| Table S2. | | | | | |
| --- | --- | --- | --- | --- | --- |
| Overweight and obesity in children with ASD (3-7 years) compared to Dutch children aged 2-21 from the Fifth National Growth Study, excluding children with ASD that use appetite inducing medication or have a migration background (Schonbeck & van Buuren, 2010). | | | | | |
|  | Children with ASD | | Reference group | | |
|  | N | % | % | Chi-square | *p* |
| *Children with ASD (3 – 7 years) using appetite inducing medication excluded* | | | | | |
| Healthy Weight | 48 | 75 | 85.9 | 45.86 | <.001 |
| Overweight | 7 | 10.9 | 12.1 |  |  |
| Obesity | 9 | 14.1 | 2 |  |  |
| *Children with ASD (3 – 7 years) from a migration background excluded* | | | | | |
| Healthy weight | 40 | 76.9 | 85.9 | 60.96 | <.001 |
| Overweight | 3 | 5.8 | 12.1 |  |  |
| Obesity | 9 | 17.3 | 2 |  |  |
