# Supplementary material for: Novel Insights into Obesity in Preschool Children with Autism Spectrum Disorder
Source: Child Psychiatry Hum Dev. 2024 Feb 1;57(1):2–9. doi: 10.1007/s10578-024-01679-1 (PMC12971737; doi:10.1007/s10578-024-01679-1)
Supplement: Supplementary file 3 — Supplementary Material 3 [file 10578_2024_1679_MOESM3_ESM.docx]

| Table S3. | | |
| --- | --- | --- |
| Correlations between BMIz of children with ASD and autism severity, eating behavior and behavior problems controlled for SES mother and ethnic background. | | |
|  | Controlling for SES | Controlling for ethnicity |
|  | BMI child | BMI child |
| *Autism severity (ADOS-2)*  Autism severity^a^ | -.08 | -.07 |
| *Child eating behavior (CEBQ)*  Food responsiveness^a^ | **.42***** | **.44***** |
| Emotional overeating^a^ | **.32**** | **.30*** |
| Enjoyment of food | **.39***** | **.36**** |
| Desire to drink | **.25*** | **.27*** |
| Satiety responsiveness | **-.25*** | -.23 |
| Slowness in eating | **-.32**** | **-.32**** |
| Emotional undereating^a^ | -.09 | -.09 |
| Food fussiness^a^ | .04 | .04 |
| *Child problem behavior (CBCL)*  Externalizing behavior problems | .10 | .10 |
| Internalizing behavior problems | .09 | .09 |
| Total behavior problems | .21 | .22* |
| Abbreviations: BMIz = Standardized Body Mass Index; ASD = Autism Spectrum Disorder; ADOS = Autism Diagnostic Observation Scale; CEBQ = Child Eating Behavior Questionnaire; CBCL = Child Behavior Checklist.. ^a^Variable was non-normality distributed, Spearman’s correlation coefficients are displayed. **p* < .05, ***p* < .01, ****p* < .001. | | |
