# Supplementary material for: Novel Insights into Obesity in Preschool Children with Autism Spectrum Disorder
Source: Child Psychiatry Hum Dev. 2024 Feb 1;57(1):2–9. doi: 10.1007/s10578-024-01679-1 (PMC12971737; doi:10.1007/s10578-024-01679-1)
Supplement: Supplementary file 4 — Supplementary Material 4 [file 10578_2024_1679_MOESM4_ESM.docx]

| Table S4. | | |
| --- | --- | --- |
| Correlations between BMI of children with ASD and parental BMI, eating behavior and SES controlled for SES and Ethnic background. | | |
|  | *Controlling for SES* | *Controlling for ethnic background* |
|  | BMI Child | BMI Child |
| *Mothers* |  |  |
| BMI^a^ | **.29*** | **.28*** |
| Emotional eating (DEBQ)^a^ | .10 | .06 |
| External eating (DEBQ) | .08 | .06 |
| Restraint eating (DEBQ)^a^ | .23 | .19 |
| Parenting stress (OBVL) | .06 | .04 |
| *Fathers* |  |  |
| BMI^a^ | .22 | .21 |
| Emotional eating (DEBQ)^a^ | <.01 | -.05 |
| External eating (DEBQ) | -.19 | -.20 |
| Restraint eating (DEBQ)^a^ | -.15 | -.19 |
| Parenting stress (OBVL) | <.01 | -.01 |
| Abbreviations: ASD = Autism Spectrum Disorder; BMI = Body Mass Index; SES = Social Economic Status; DEBQ = Dutch Eating Behavior Questionnaire; OBVL = Parenting Stress Questionnaire; SES = Social Economic Status.  ^a^Variable was non-normally distributed, Spearman’s correlation coefficients are displayed. **p* < .05, ***p* < .01, ****p* < .001. | | |
